# Liposarcoma of the pelvic fossa masquerading as hematoma: a rare case report and its surgical management

**DOI:** 10.1093/jscr/rjab120

**Published:** 2021-06-12

**Authors:** Maria Zarokosta, Aikaterini Foutsitzi, Spyridon Roditis, Vasilissa Karanasiou, Eirini Nannou, Theodoros Mariolis-Sapsakos

**Affiliations:** Anatomy and Histology Laboratory, Nursing School, University of Athens, Athens, Greece; University Department of Surgery, General and Oncologic Hospital of Kifissia “Agii Anargiri”, Athens, Greece and Anatomy and Histology Laboratory, Nursing School, University of Athens, Athens, Greece; Anatomy and Histology Laboratory, Nursing School, University of Athens, Athens, Greece; University Department of Surgery, General and Oncologic Hospital of Kifissia “Agii Anargiri”, Athens, Greece and Anatomy and Histology Laboratory, Nursing School, University of Athens, Athens, Greece; Anatomy and Histology Laboratory, Nursing School, University of Athens, Athens, Greece; Anatomy and Histology Laboratory, Nursing School, University of Athens, Athens, Greece; Anatomy and Histology Laboratory, Nursing School, University of Athens, Athens, Greece; Anatomy and Histology Laboratory, Nursing School, University of Athens, Athens, Greece; University Department of Surgery, General and Oncologic Hospital of Kifissia “Agii Anargiri”, Athens, Greece and Anatomy and Histology Laboratory, Nursing School, University of Athens, Athens, Greece

## Abstract

Liposarcomas constitute rare malignant tumors of the soft tissue, with wide anatomical distribution. The prompt diagnosis of a liposarcoma is extremely challenging since these tumors tend to remain asymptomatic, until they grow enough to displace adjacent anatomical structures. In the presented case, a 55-year-old Caucasian male proceeded to our institution complaining about irreducible swelling of the right iliac fossa and constant discomfort, over the course of a year. His medical history revealed injury of the right groin and pelvis a year ago. The diagnosis was ilioinguinal liposarcoma masquerading as hematoma, due to the previous injury. The patient underwent primary complete tumor resection, and the operation was uneventful. The essential diagnostic and surgical steps for the management of a liposarcoma, mimicking a hematoma are meticulously described.

## INTRODUCTION

Liposarcomas constitute rare malignant tumors of the soft tissue, with wide anatomical distribution [1]. The prompt diagnosis of a liposarcoma is extremely challenging since these tumors tend to remain asymptomatic, until they grow enough to displace adjacent anatomical structures [2]. However, a miss correct diagnosis has potentially fatal consequences [3]. Herein, the present manuscript aims to highlight a rare case of an ilioinguinal liposarcoma masquerading as a hematoma and its surgical management.

## CASE REPORT

**Figure 1:**
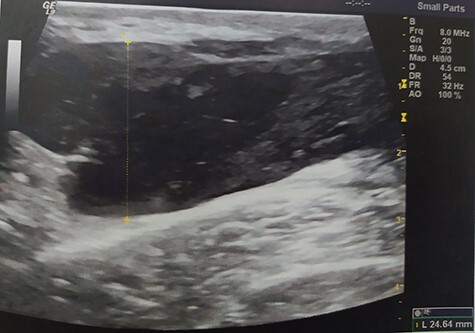
Pelvic ultrasound indicating an acute hematoma into the fatty tissue of the right iliac fossa.

**Figure 2:**
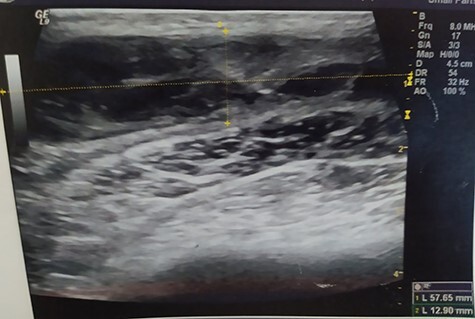
Pelvic ultrasound indicating an acute/nonorganized hematoma into the fatty tissue of the right iliac fossa, with thrombotic features.

A 55-year-old Caucasian male proceeded to our institution complaining about irreducible swelling of the right iliac fossa and constant discomfort, over the course of a year. His medical history revealed that he slapped against a blunt surface of a furniture a year ago and injured his right groin and pelvis. Due to the accident and the subsequent painful swelling, the patient underwent immediately a pelvic ultrasound the detected an acute hematoma with a diameter of 52 mm into the fatty tissue of the right pelvic fossa **(**Fig. 1**)**. Ten months after the accident, due to the irreducible swelling, the patient underwent another ultrasound revealing a nonorganized hematoma with thrombotic features (sized as 25 × 60 × 58 mm) **(**Fig. 2**)**.

**Figure 3:**
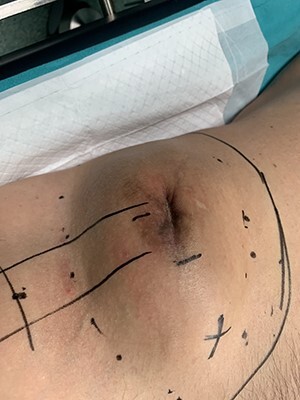
Patient’s appearance on physical examination. Swelling of the right ilioinguinal area and palpation of a large, hard mass into the right iliac fossa.

**Figure 4:**
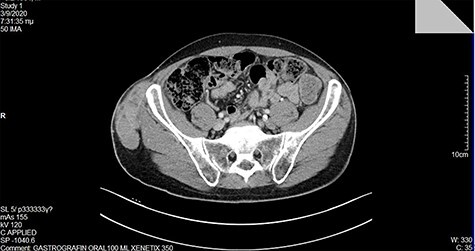
A large lipomatous mass detected in the MRI.

At our institution, on clinical examination, surgeons verified the swelling of the ilioinguinal aera and detected at tumescent, palpable and hard mass of the right pelvic fossa **(**Fig. 3**)**. Lab examinations were unremarkable. Subsequent pelvic magnetic resonance imaging (MRI) **(**Fig. 4**)** and computed tomography (CT) scan identified a tumescent lipomatous tumor, without the presence of any metastatic lesions. Guided biopsy indicated the presence of an ilioinguinal liposarcoma located into the fatty tissue of the right iliac fossa. Following these, the interdisciplinary team decided to schedule a primary complete curative resection of the tumor (R0).

**Figure 5:**
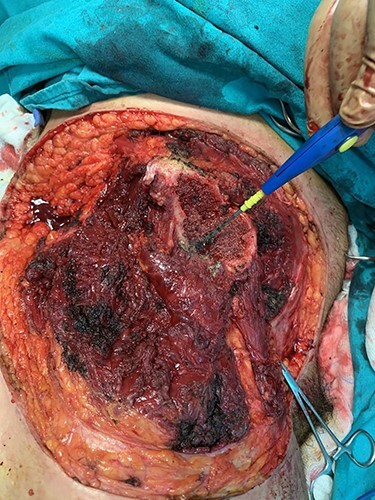
Liposarcoma complete resection.

**Figure 6:**
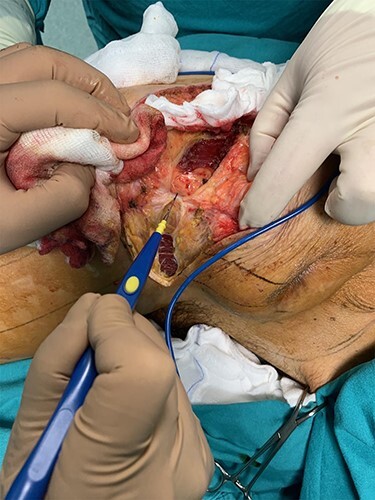
Elective resection of the right iliac crest and part of the right gluteus maximus muscle.

**Figures 7:**
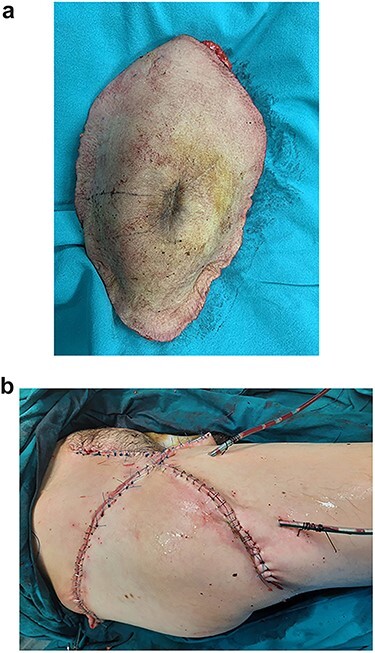
The resected liposarcoma with the adjacent tissues, for the elimination of the potentiality of local recurrence.

During the operation, surgeons exposed meticulously the liposarcoma that was utterly removed, and performed elective resection of the right iliac crest, the upper part of the sartorius muscle, part of the gluteus maximus muscle, and part of the muscles of the anterior abdominal wall in order to eliminate the potentiality of local recurrence of the liposarcoma **(**Figs. 5–7**)**. The operation was uneventful, and two drainages were placed into the pelvic fossa. The drainages were removed the sixth postoperative day when the patient was finally discharged with instructions. The pathology report documented a well-differentiated liposarcoma of the right pelvic fossa. Finally, at the 6-month follow-up, the patient had no complication or recurrent lesions and oncologists declared that adjuvant therapy was not essential.

## DISCUSSION

Liposarcomas are rare malignancies, that are classified into five subtypes according to their histologic features: (i) well-differentiated, (ii) dedifferentiated, (iii) myxoid, (iv) pleomorphic and (v) mixed [4]. Although rare, liposarcoma constitutes the second most frequent soft tissue tumor, with the prevalence of 10–35% of these malignancies [5].

Liposarcomas typically present into the retroperitoneum and the tight, arising from the fatty tissue, the spermatic cord or even the omental tissue [3]. However, their anatomical distribution is wide, including the mediastinum, the iliac fossa and the thorax [2].

The diagnosis of liposarcomas may be hindered, since they remain asymptomatic until they grow enough to apply pressure to adjacent structures, with potentially fatal consequences [2, 3]. Nevertheless, imaging evaluation (MRI and CT-scan) may be extremely helpful, in addition to guided biopsy, as in the presented case [5].

Indeed, the diagnosis may be extremely challenging, especially when a small tumor component is ducked under a hematoma, as in the presented case. The present manuscript describes an ilioinguinal liposarcoma masquerading as a hematoma of the pelvic fossa. Although rare as a liposarcoma manifestation, Lin *et al.* also reported an extrapleural liposarcoma obscured by the presence of hematoma due to previous injury of the thorax [2]. Therefore, when surgeons encounter an enlarging or irreducible hematoma and soft tissue swelling, should always suspect the presence of a ‘hidden’ liposarcoma.

Finally, treatment of liposarcomas typically includes meticulous surgical resection, as in the present case, and adjuvant radiotherapy or chemotherapy when appropriate [6]. Despite the operation and the elective resection of adjacent to the tumor structures, patients’ tactical follow-up is of paramount clinical significance, since liposarcomas are malignancies that tend to present with recurrent lesions after the surgery [4].

Conclusively, surgeons’clinical suspicion regarding liposarcoma, is fundamental and crucial when encountering constant swelling and irreducible hematomas of the soft tissue, despite their rarity. Surgeons deep knowledge in addition to emphasis on patient’s medical history is a cornerstone for the prompt diagnosis of liposarcoma, and its successful and safe complete surgical resection.
